# Freedom

**Published:** 1890-03-01

**Authors:** 


### Words of Consolation.

### FREEDOM.

*For Reading to the Sick.*

Slavery is a condition of life which raises compassion in the heart of every feeling person. Philanthropists have striven in the past to do away with this horrible traffic in human beings, at the cost of their own property and lives, and in a measure have been successful. Travellers, however, tell us that unhappily in many parts of Africa the brutal trade still survives in its worst form. The harmless inhabitants of those regions are lulled into security by kindness and presents, from the Arab merchants, who collect ivory and other articles of merchandise, and when they least expect it a whole village is seized upon it once; men, women, and children are laden with heavy burdens too great for their strength, and driven with whips and tortures indescribable to the sea-shore. Hundreds fall and die on the road, and the wretched survivors are sold for slaves. Can anything be sadder or more awful? It seems to us the very height of misery and distress.

But there is a worse slavery even than that. It is the bondage of the soul to Satan. He, the Prince of this World, gives men ease and pleasure and the good things of life for a season, they are happy and secure. Suddenly destruction comes upon them like a sharp sword and they are afraid. They have become slaves to their own passions and lusts, their "pleasant sins" are turned into whips and scorpions to drive them to despair. They see how they have fallen into such a state. They have listened to the Tempter and taken to themselves his promises as Eve did, and now their eyes are opened, they know to what a pass he has brought them. They long to be free from Satan's yoke, but are powerless to help themselves. They have despised the light burden and easy yoke of the Lord, who bought them with His precious blood, and who in loving accents says to all "learn of me." To be free does not mean license to run riot through all the Ten Commandments, defying both God and man, but it is the power which only God can give us, of holding ourselves well in hand, to have control over our tempers and passions, to "Live by law, acting the law we live by, and because right is right to follow right," without regard to the consequences of our actions. Satan's yoke crushes and destroys the soul. Christ's yoke strengthens and sets us free. He takes us out of the hand of the enemy, and sets our feet in a large room.

Are you too slothful to break away from your chains? Gird yourselves up, and think of what men have done and suffered in old times for the love of their country and their rights. Cannot you do the same for greater rights—the right of calling yourself one of God's people? Those others strove for an earthly city and country, but your reward will be a heavenly one. Call then upon your Lord and Saviour to help you. He has ransomed you and all mankind by His precious blood, and to do that bore the spite and malice of of His enemies, the buffetings and scourgings of wicked men, and the agony of His Cross and Passion. If He bore all this for you, and made such sacrifices for your benefit, can you be so blind and foolish as to go on sighing in your slavery without making an effort to be free? Your efforts would, indeed, be useless without Christ's help; you are in the power of the Strong Man, but when a stronger than He cometh your victory is certain.

